# Supplementary material for: Shaping the genome via lengthwise compaction, phase separation, and lamina adhesion
Source: Nucleic Acids Res. 2022 Apr 14;50(8):4258–71. doi: 10.1093/nar/gkac231 (PMC9071446; doi:10.1093/nar/gkac231)
Supplement: gkac231_Supplemental_File [file gkac231_supplemental_file.pdf]

Supplementary Material: Shaping the Genome via Lengthwise Compaction, Phase Separation, and Lamina Adhesion

Sumitabha Brahmachari,[1] Vinícius Contessoto,[1] Michele Di Pierro,[2] and José N. Onuchic[1]

[1] *Center for Theoretical Biological Physics, Rice University, Houston, TX 77005, USA*

[2] *Department of Physics, Northeastern University, Boston, MA 02115, USA*

I. SIMULATION METHODS

Contact function

The contact function is a sigmoidal function that cuts off interactions between distant monomers, and this is used to calibrate the contact probability between genomic segments [1]. The contact function $f(r_{i,j})$ is defined as:

$$f(r_{i,j}) = \frac{1}{2}\left(1 + \tanh\left[\mu(r_c - r_{i,j}\right]\right) \tag{1}$$

where $\mu = 3.22$ ad $r_c = 1.78$ are used following previous calibration with experimental Hi-C maps [1]. Note, the qualitative results discussed in the main text are not sensitive to small changes in these parameters.

Interaction potential

Homopolymer

The homopolymer potential (U_{HP}) models a generic bead-spring polymer in which each bead represents a genomic segment containing 20-50 Kb of DNA, where chromosome topology fluctuations are controlled by using an energy barrier. This potential consists of the following five terms, U_{FENE} , U_{Angle} , U_{hc} and, U_{sc}:

$$U_{HP} = \sum_{i \in \{\text{Loci}\}} U_{\text{FENE}}(r_{i,i+1}) + \sum_{i \in \{\text{Loci}\}} U_{hc}(r_{i,i+1}) + \sum_{i \in \{\text{Angles}\}} U_{\text{Angle}}(\theta_i) + \sum_{\substack{i,j \in \{\text{Loci}\} \\ j > i+2}} U_{sc}(r_{i,j}),$$

where,

$$U_{\text{FENE}}(r_{i,j}) = \begin{cases} -\frac{1}{2} k_b R_0^2 \ln\left[1 - \left(\frac{r_{i,j}}{R_0}\right)\right] & r_{i,j} \leq R_0 \\ 0 & r_{i,j} > R_0 \end{cases}$$

U_{FENE} (Finite Extensible Nonlinear Elastic potential) is the bonding term applied between two consecutive monomers of a chromosome.

$$U_{hc}(r_{i,j}) = \begin{cases} 4\epsilon\left[\left(\frac{\sigma}{r_{i,j}}\right)^{12} - \left(\frac{\sigma}{r_{i,j}}\right)^{6} + \frac{1}{4}\right] & r_{i,j} \leq \sigma 2^{\frac{1}{6}} \\ 0 & r_{i,j} > \sigma 2^{\frac{1}{6}} \end{cases}$$

$U_{hc}(r_{i,j})$ is the hard-core repulsive potential, include to avoid overlap between the bonded nearest neighbor monomers.

$$U_{\text{Angle}}(\theta_i) = k_a[1 - \cos(\Theta_i - \theta_0)],$$

a three-body potential applied to all connected three consecutive monomers of a chromosome, where Θ_i is the angle defined by two vectors $\vec{r}_{i,i+1}$ and $\vec{r}_{i,i-1}$, and $\theta_0 = 0$ is the equilibrium angle.

The non-bonded pairs is defined by a soft-core repulsive interaction in the following form:

$$U_{sc}(r_{i,j}) = \begin{cases} \frac{1}{2}E_{\text{cut}}\left[1 + \tanh\left(\frac{2U_{LJ}(r_{i,j})}{E_{\text{cut}}} - 1\right)\right] & r_{i,j} \leq r_0 \\ U_{LJ}(r_{i,j}) & r_0 \leq r_{i,j} \leq \sigma 2^{\frac{1}{6}} \\ 0 & r_{i,j} > \sigma 2^{\frac{1}{6}} \end{cases}$$

The expression U_{LJ} correspond to the Lennard-Jones potential:

$$U_{LJ}(r_{i,j}) = 4\epsilon\left[\left(\frac{\sigma}{r_{i,j}}\right)^{12} - \left(\frac{\sigma}{r_{i,j}}\right)^{6} + \frac{1}{4}\right],$$

capped off at a finite distance, thus allowing for chain crossing at finite energetic cost. The parameter r_0 is chosen as the distance at which $U_{LJ}(r_{i,j}) = \frac{1}{2}E_{cut}$. Note that this potential is applied across all non-neighboring monomers of the system.

Lengthwise Compaction

We implement lengthwise compaction of the polymer as a sum of two terms:

$$U_{LC} = \sum_{\substack{i,j\in\{\text{Cis-loci}\}\\ j>i+3}} -\frac{A_L}{\sqrt{|i-j|}} - A_S\exp(-|i-j|/\ell) \tag{2}$$

where $\ell = 10$ is the characteristic length of short-range compaction. The potential is applied between intra-chain monomers that are more than 3 monomers apart, and underlies a looping tendency. The first term with amplitude $A_L > 0$ controls the long range compaction, while the second term with amplitude $A_S > 0$ controls the short-range compaction. Note that the intensity of lengthwise compaction depends on the genomic distance between the two loci, and that this potential does not act across chromosomes. Different values of A_L and A_S

leads to the structural phenotypes described in the main text: SAW ($A_L = 0.05$, $A_S = 0.05$), Globular ($A_L = 0.4$, $A_S = 0.05$), String-like ($A_L = 0.05$, $A_S = 0.4$), and Rope-like ($A_L = 0.4$, $A_S = 0.4$).

Phase Separation

The potential associated with phase separation is self-adhesion among monomers. There is a generic adhesion between any two monomers of intensity $\chi = -0.2$. This implies whenever two monomers come within interaction distance of one another the energy of the system lowers by χ (where the units are in simulation energy scale $\epsilon = k_BT$). The centromere monomers adhere to other centromere monomers with an enhanced adhesive interaction χ_C. The interaction potential is represented as follows:

$$U_{PS} = \sum_{i,j \in \{\text{all loci}\}} \chi + \sum_{i,j \in \{\text{centromere loci}\}} (\chi_C - \chi) \tag{3}$$

Lamina Adhesion

We place static monomers at a distance R_0 from the center of mass of the genome, such as to form a rigid spherical shell of radius R_0 encapsulating the genome. The numerical value of R_0 is decided from the requirement of physiological volume fraction ($\phi \approx 0.1$) of the genome inside the nucleus: $R_0 = \sigma(N/(8\phi))^{1/3}$, where N is the total number of monomers in the genome, and $\sigma = 1$ is the monomer diameter.

While all the lamina beads and genome beads experience a soft-core repulsion, a randomly selected subgroup of 30% of the surface beads interact favorably with the centromere with an interaction strength $\chi_L \leq 0$. The nuclear envelope contains many elements like the nuclear pores that cover a significant portion of the surface area that are not adhesive to the chromosome segments. Given the genome volume fraction is about 10%, using a sub population of surface beads made the competition between phase separation and lamina adhesion occur for similar values of χ_L and χ_C. The lamina interaction potential may be expressed as follows:

$$U_{Lam} = \sum_{\substack{i \in \{\text{Loci}\} \\ j \in \{\text{Lamina}\}}} U_{sc}(r_{i,j}) + \sum_{\substack{i \in \{\text{centromere}\} \\ j \in \{\text{adh-lamina}\}}} \chi_L \tag{4}$$

where 'adh-lamina' refers to the subgroup of lamina monomers that adhere to the centromere.

II. TRAJECTORY ANALYSIS

The analysis were done on an ensemble of simulated trajectories. We simulated each parameter set for 2×10^7 time-steps ($dt = 10^{-3}$), and generated 10 replicas of each trajectory with randomized initial configurations. We use high temperature annealing of the homopolymer model to generate many random structures that are used to initialize the simulations. We then neglect the initial 10^6 time steps from our analysis, to ensure the steady-state nature of our trajectories (Fig. S13).

Contact probability matrices

Contact probability matrices, the analogue of HiC-maps, are calculated using the contact function. For every frame, we compute the pairwise distance between monomers and then use the contact function to convert the distance into contact probability, following our previous approach [1]. All the snapshots corresponding to a parameter set are then averaged to generate the contact maps shown in the main and supplementary figures.

Principal component eigenvector

The outer product of the contact probability matrices were used to generate the correlation matrix. The eigenvector corresponding to the largest eigenvalue is the principal component eigenvector shown along with the contact maps. These principal components have been used to annotate compartments in HiC experiments [1, 2].

Voronoi tessellation: Territory strength and trans-centromere contacts

One snapshot of the trajectory can be considered to be a discrete distribution of points in 3D space. Using Voronoi tessellation, the empty space between points maybe filled by non-overlapping polyhedrons, each encapsulating one bead. Each surface of a polyhedron is such that it is a plane perpendicularly bisecting the line connecting a bead to its neighbor. The number of surfaces of the polyhedron defines the number of neighbors for the encapsulated

bead. We use the python package: Scipy.spatial.Voronoi [3] to compute the Voronoi diagram in our simulated trajectories.

Using this scheme, we identify neighbors of a monomer, and then classify the ratio of number of intra-chain contacts to the total number of contacts per chain as the strength of the chromosome territory. Similarly, the proportion of trans-centromere contacts is computed from the ratio of number of inter-chromosome centromere contacts to the total number of centromere-centromere contacts.

Shape analysis: Gyration tensor

Attributes of chromosome shape, like the radius of gyration and the shape anisotropy, are calculated using the gyration tensor $\mathcal{G}$, defined as follows for a set of coordinates:

$$\mathcal{G} = \begin{bmatrix} \sum_i (x_i - x_{cm})^2 & \sum_i (x_i - x_{cm})(y_i - y_{cm}) & \sum_i (x_i - x_{cm})(z_i - z_{cm}) \\ \sum_i (x_i - x_{cm})(y_i - y_{cm}) & \sum_i (y_i - y_{cm})^2 & \sum_i (y_i - y_{cm})(z_i - z_{cm}) \\ \sum_i (x_i - x_{cm})(z_i - z_{cm}) & \sum_i (y_i - y_{cm})(z_i - z_{cm}) & \sum_i (z_i - z_{cm})^2 \end{bmatrix} \quad (5)$$

Here the i-sum is over all the monomers of the polymer whose shape we are interested in, and (x_{cm}, y_{cm}, z_{cm}) is the center of mass of the polymer. Once this matrix is computed for a given snapshot, we compute the eigenvalues of the gyration tensor $\lambda_1, \lambda_2, \lambda_3$ (note, $\lambda_1 \geq \lambda_2 \geq \lambda_3 > 0$) and then calculate the shape descriptors as follows [4]:

$$R_g = \sqrt{\lambda_1 + \lambda_2 + \lambda_3} \quad (6)$$

$$c = \lambda_2 - \lambda_3 \quad (7)$$

$$\kappa^2 = \frac{3(\lambda_1^2 + \lambda_2^2 + \lambda_3^2)}{2(\lambda_1 + \lambda_2 + \lambda_3)^2} - \frac{1}{2} \quad (8)$$

where R_g is the radius of gyration. c is the acylindricity, which is lower if there is cylindrical symmetry in the conformation. And, κ^2 is the relative shape anisotropy that is bound between 0 and 1, and is higher for anisotropic structures.

Hierarchical clustering: Number of centromere clusters

Clusters of centromeres and telomeres were defined using the hierarchical clustering algorithm via constructing a dendrogram. At the first step, each centromere monomer is

considered a cluster, this is the largest possible number of clusters in the genome. Iteratively, clusters are merged, following a condition that the shift in the centroid of the cluster due to the merge is smaller than a cut-off value. We choose this cut-off to be twice the radius of gyration of the cluster (note, using a slightly different value, like three times the radius of gyration, does not change the qualitative nature of our results). When merging two clusters is shifts the centroid to larger than the cut-off, those two clusters are identified as two individual clusters. We use the python module Scipy.cluster [3] to implement hierarchical clustering. The number of clusters obtained from every snapshot is then plotted using a histogram.

Radial density distribution

Radial distribution of monomers is calculated from the snapshots. The volume occupied by the genome is divided into concentric shells centered at the centroid of the genome, then the number of monomers is each cell is counted. To obtain the density, the number is divided by the volume of the shell.

Gauss linking number: inter chromosome entanglements

The Gauss linking number between two chromosomes, counts the number of signed crossings between the them, and measures their entanglement. We use the "method 1a", as prescribed in Ref. [5], to compute the linking number. Since linking number is defined only for closed curves, we simply connect the two ends of each chromosome to close the curve during our computation. We calculate entanglement for each snapshot of an ensemble and then plot the histogram of linking number values, showing the distribution of linking numbers.

Simulation snapshots

The simulation snapshot images are made using the VMD software [6].

III. SUPPLEMENTARY FIGURES

See Figures S1-S13.

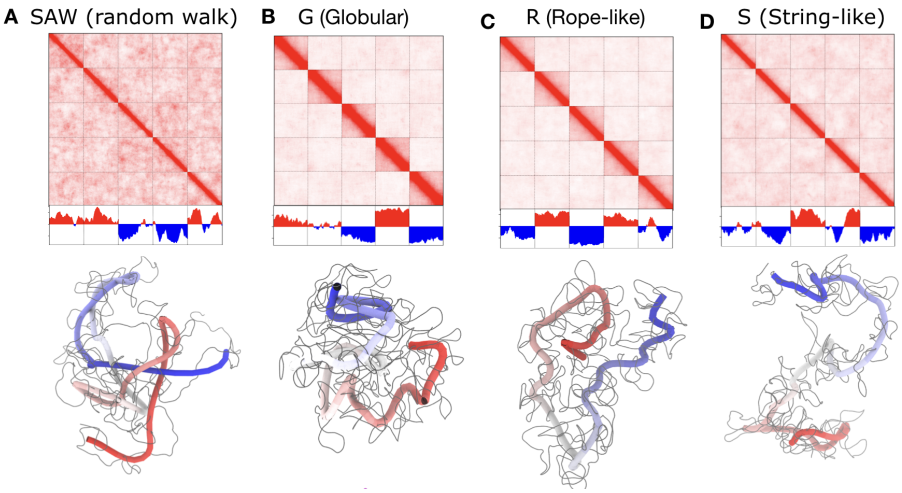

FIG. S1. **Lengthwise compaction controls chromosome territories.** Contact maps corresponding to (A) SAW, (B) Globular (C) Rope, (D) String phenotypes are shown for chromosomes with $\chi_C = \chi_T = 0$. Below each contact map are representative structures. A chromosome when renormalized by 20 monomers, shows the underlying backbone. This backbone is shown as a tube in blue-to-red coloration.

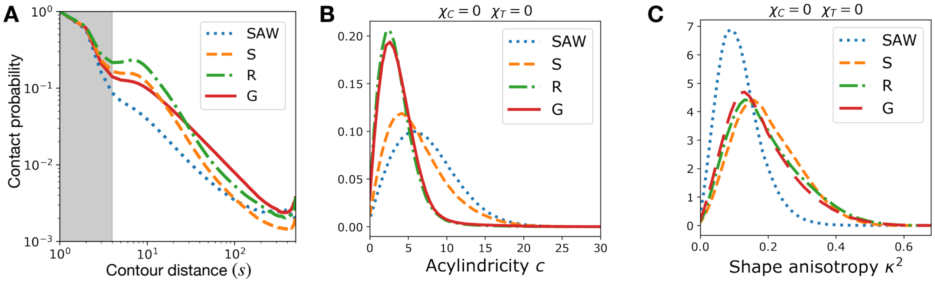

FIG. S2. **Lengthwise compaction shapes chromosomes.** (A) Contact probability of intra-chromosome segments as a function of contour distance. The contact probability of a monomer with its neighbors, up to 3 or 4 monomers are mainly controlled by the angle-restrain part of of the homopolymer potential. The contact probability between the monomers beyond about 5 monomers is controlled by lengthwise compaction. (B) Acylindricity and (C) Shape anisotropy calculated from the Gyration tensor for chromosomes with different lengthwise compaction intensity: SAW, Globular Rope, and String phenotypes. Higher lengthwise compaction introduces cylindrical asymmetry in chromosomes structure.

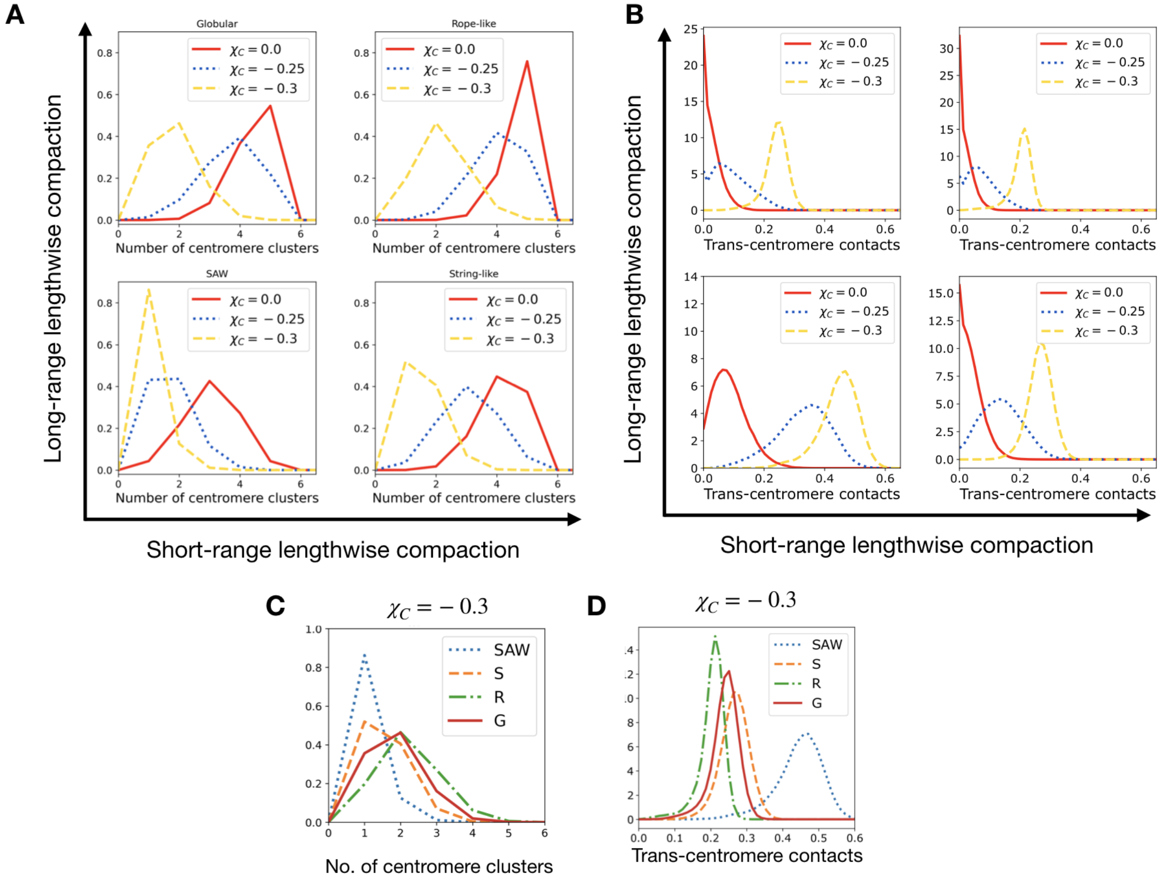

FIG. S3. **Centromere clustering is enhanced by centromere self-adhesion and counteracted by lengthwise compaction.** (A) Number of centromere clusters and (B) Proportion of trans-centromere contacts, shown under different centromere self-adhesive interaction χ_C, for various long- and short-range lengthwise compacted chromosomes. (C) Number of centromere clusters and (D) Trans-centromere contacts under $\chi_C = -0.3$ for various lengthwise compaction.

[1] Di Pierro M et al. "Transferable model for chromosome architecture." *Proc Natl Acad Sci USA* **113** 43 (2016)

[2] Aiden EL, et al "Comprehensive Mapping of Long-Range Interactions Reveals Folding Principles of the Human Genome" *Science* **33292** (2009)

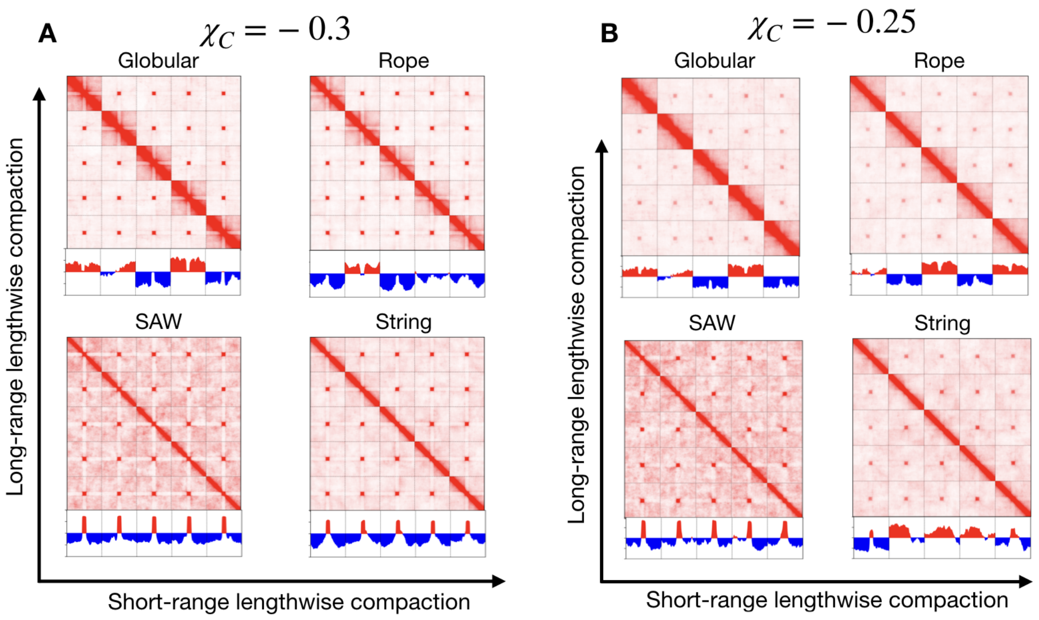

FIG. S4. **Contact matrices for different lengthwise compaction with centromere adhesion.** Contact matrices and principal eigenvectors for SAW, G, R and S states with (A) strong ($\chi_C = -0.3$) and (B) moderate ($\chi_C = -0.25$) centromere adhesion.

[3] Virtanen, Pauli, et al., "SciPy 1.0: Fundamental Algorithms for Scientific Computing in Python", *Nature Methods* **17** 261-272 (2020),

[4] Doros N. Theodorou and Ulrich W. Suter, "Shape of unperturbed linear polymers: polypropylene" *Macromolecules* **18** (6), 1206-1214 (1985)

[5] Klenin K, and Langowski J "Computation of writhe in modeling of supercoiled DNA" *Biopoly.* **54** (2000)

[6] Humphrey, W., Dalke, A. and Schulten, K., "VMD - Visual Molecular Dynamics", *J. Molec. Graphics* **14** 33-38 (1996).

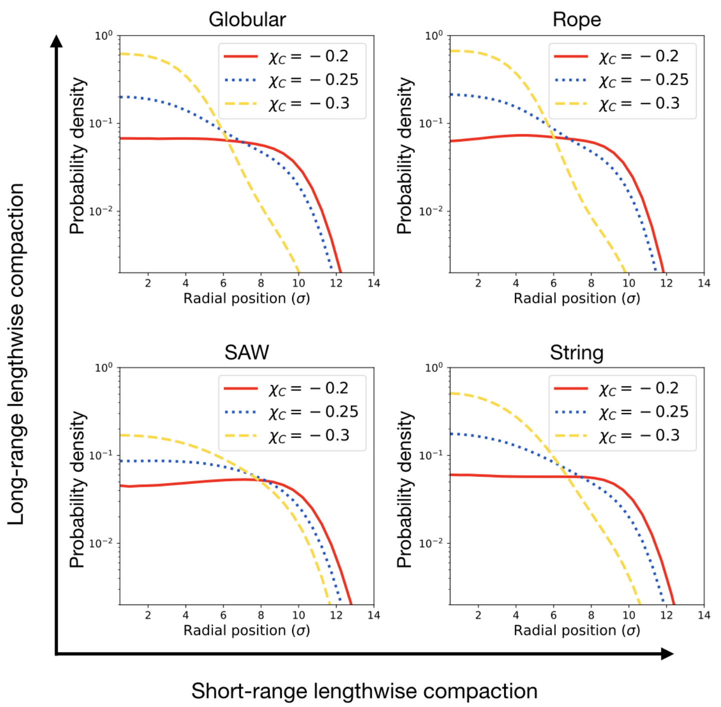

FIG. S5. **Compact centromeres with strong self-adhesion move to the center of the nucleus.** Radial probability density of centromeres under various self-adhesion and lengthwise compaction shown. Note that the compact centromeres corresponding to Globular, Rope, and String-phenotypes when strongly adhere to other centromeres tend to localize near the center of the nucleus.

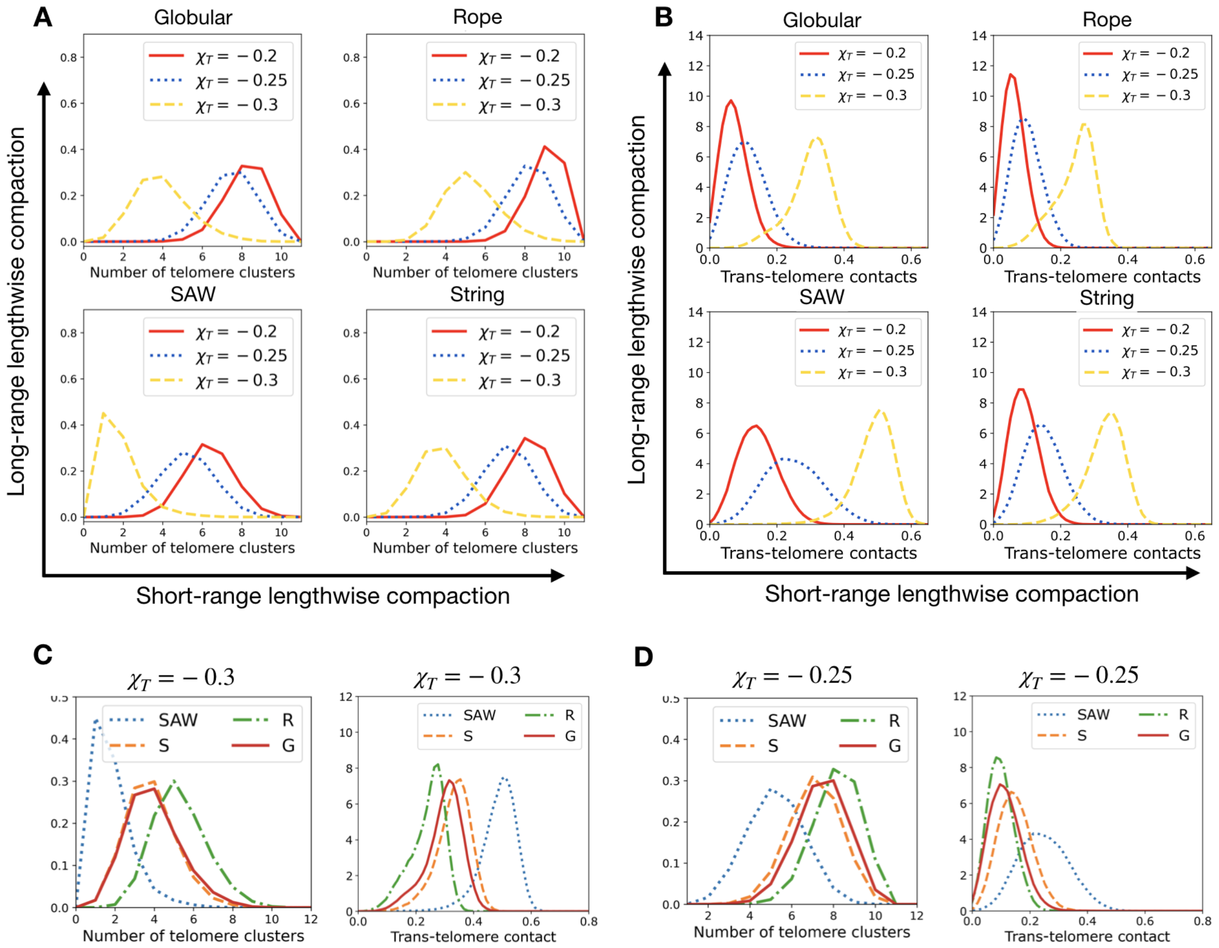

FIG. S6. **Telomere clustering is enhanced by telomere self-adhesion and counteracted by lengthwise compaction.** (A) Number of telomere clusters (B) Proportion of trans-telomere contacts shown for various telomere adhesive interactions χ_T and under different lengthwise compaction. Number of telomere clusters and trans-telomere contacts for (C) $\chi_T = -0.3$ and (D) $\chi_T = -0.25$ compared for SAW, G, R and S-states.

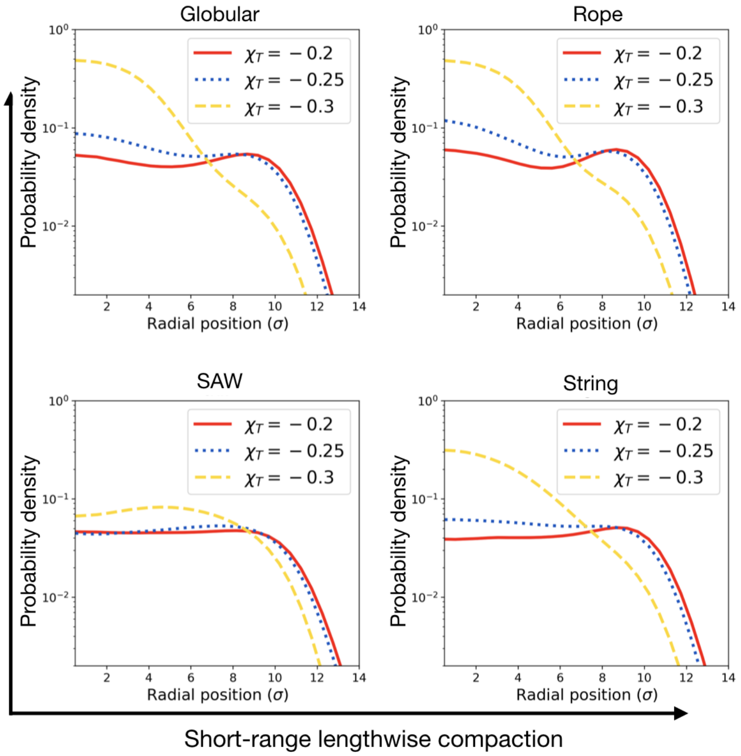

FIG. S7. **Radial density profile of telomeres.** Radial probability density of telomeres under various self-adhesion and lengthwise compaction shown. Telomeres without preferential self-adhesion reside near the periphery when lengthwise compaction is high. This is likely due to the stiffness of the chromosome backbone that tends to place its ends near the periphery. However, upon strong self-adhesion, the telomeres move to the interior, much like the centromeres under similar conditions.

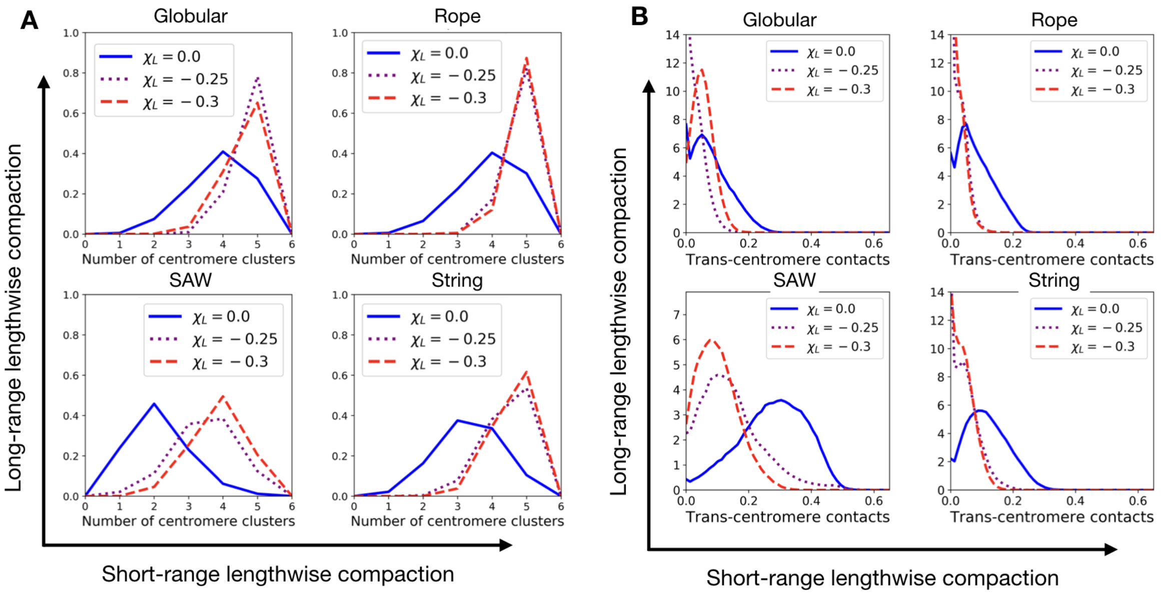

FIG. S8. **Lamina adhesion of centromeres counteract their clustering.** (A) Number of centromeres and (B) Proportion of trans-centromere contacts for moderate ($\chi_C = -0.25$) centromere self-adhesion but various lamina-tethering intensity χ_L, showing higher χ_L increases the number of centromere clusters, and reduces the propensity of inter-centromere contacts.

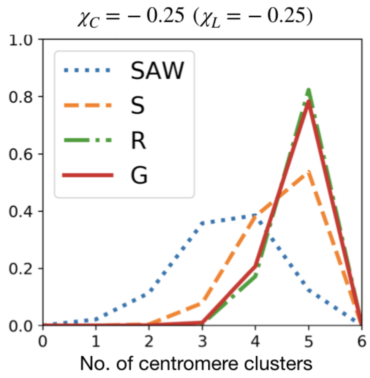

FIG. S9. **Lengthwise compaction counteracts centromere clustering in presence of lamina adheison.** Number of centromere clusters for chromosomes with moderate lamina adhesion of centromeres ($\chi_L = -0.25$) and centromere self adhesion ($\chi_C = -0.25$) under various lengthwise compaction.

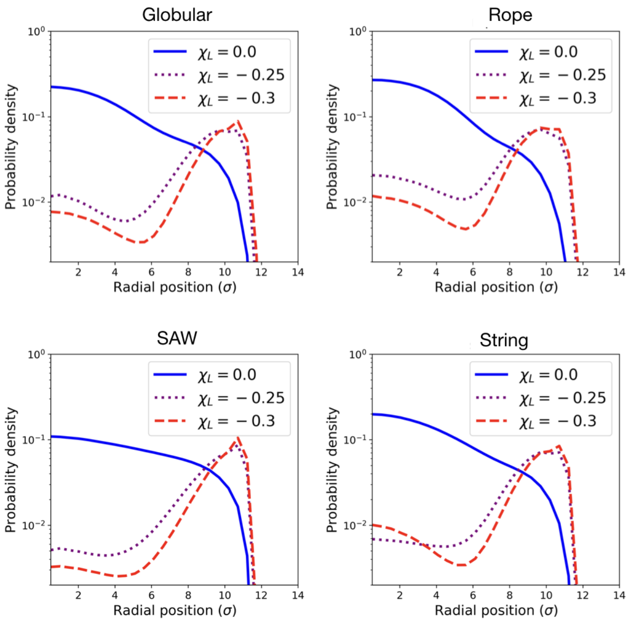

FIG. S10. **Radial density profile of centromeres with lamina adhesion.** Radial density profile is shown for centromeres with self-adhesive intensity $\chi_C = -0.25$. Centromeres tend to move to the periphery when interacting favorably with the lamina.

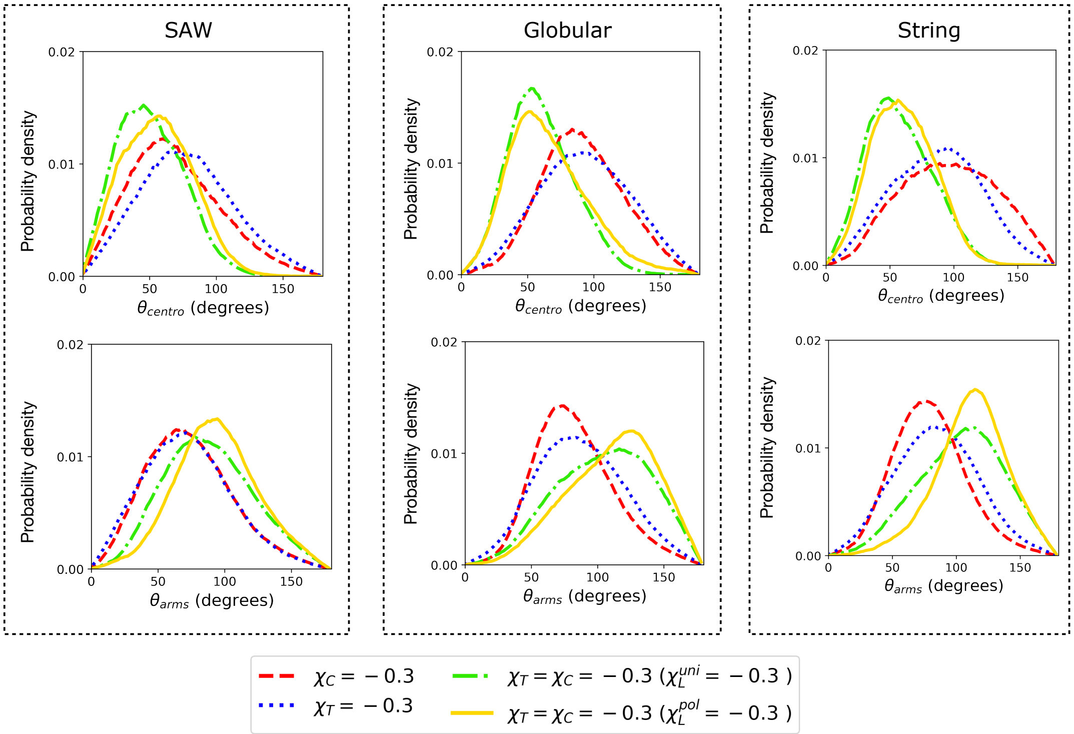

FIG. S11. **In presence of strong lamina tethering of centromeres and telomeres, lengthwise compaction may aid fold over.** Fold over angles: θ_{centro} and θ_{arm} for various cases, legend is the same as the Fig. 4D,E. The three panels correspond to different lengthwise compaction states, the rope-like phenotype is shown in Fig. 4.

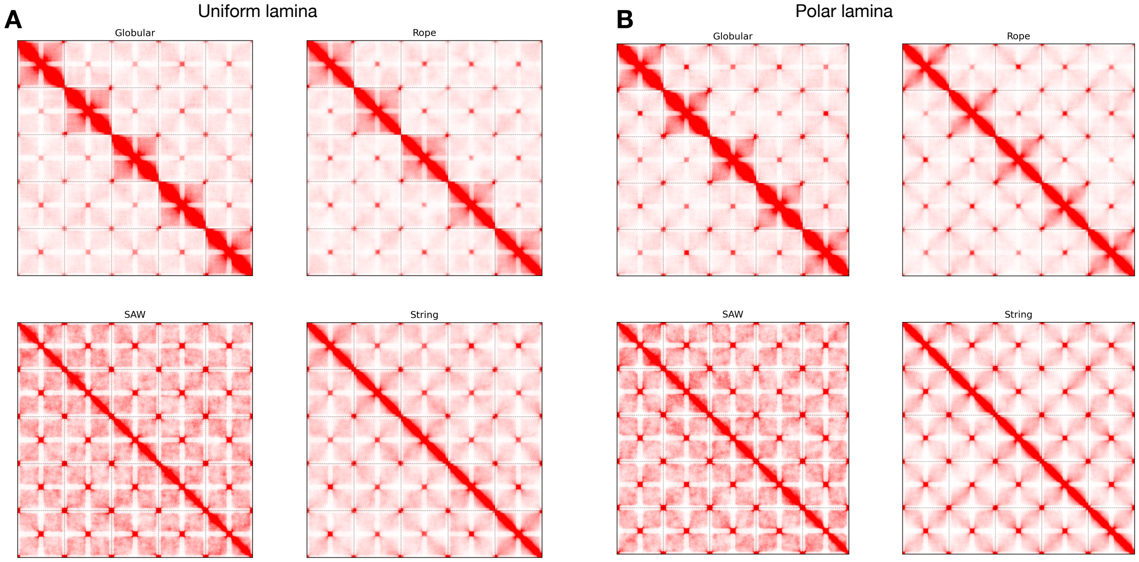

FIG. S12. **Contact maps showing fold over for polar and uniform lamina interactions.** The contact maps for (A) uniform and (B) polar lamina interactions of strnegth $\chi_L = -0.3$ for various lengthwise compaction states. All the panels correspond to strong centromere and telomere adhesion ($\chi_C = \chi_T = -0.3$).

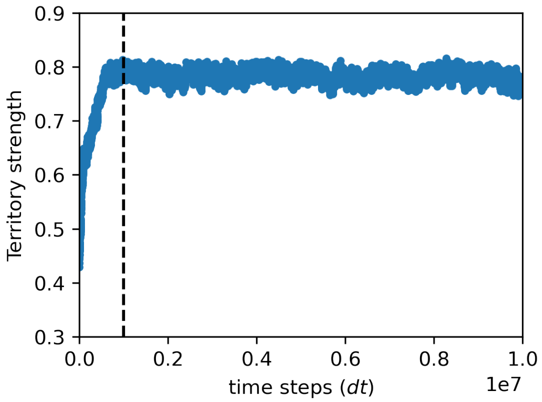

FIG. S13. **Steady state trajectories.** The territory signal is shown for a single replica as a function of simulation time. The system was initialized as a SAW (hence the low territory at zero time), but simulated under the potential for G. The system reaches its steady state corresponding to G chromosomes in less than 10^6 time steps. We exclude the initial one million time steps from our analysis (dashed line), to ensure that the memory of the initial configuration is completely lost, and we are capturing the steady state.
